# SIRT6 deficiency causes ovarian hypoplasia by affecting *Plod1*‐related collagen formation

**DOI:** 10.1111/acel.14031

**Published:** 2023-11-07

**Authors:** Liyuan Li, Rui Hua, Kaiqiang Hu, Huiling Chen, Yuemiao Yin, Xiaojin Shi, Kezheng Peng, Qing Huang, Ying Qiu, Xue Li, Qingfei Liu, Shangfeng Liu, Zhao Wang

**Affiliations:** ^1^ Protein Science Key Laboratory of the Ministry of Education, School of Pharmaceutical Sciences Tsinghua University Beijing PR China; ^2^ Tsinghua‐Peking Center for Life Sciences Beijing PR China; ^3^ School of Medicine Tsinghua University Beijing PR China; ^4^ Department of Stomatology, Huashan Hospital Fudan University Shanghai PR China

**Keywords:** collagen, estrogen biosynthesis, ovarian follicle, *Plod1*, SIRT6

## Abstract

SIRT6 is a key member of the mammalian sirtuin family of conserved nicotinamide adenine dinucleotide (NAD^+^)‐dependent deacetylases. Previous studies have shown that SIRT6 can regulate metabolism, DNA damage repair and aging. Ovarian aging process usually share similar mechanisms with general aging, which is characterized by decreases in both numbers of ovarian follicles and the quality of oocytes. It is reported that the expression level of SIRT6 was significantly decreased in the ovaries of aged mice, and the level of SIRT6 was positively correlated with ovarian reserve, indicating that SIRT6 may be potential markers of ovarian aging. However, its biological roles in follicular development are still unclear. Here, we explored the effect of SIRT6 on follicular development and found that ovarian development was interrupted in SIRT6 knockout (KO) mice, leading to disruptions of puberty and the estrus cycle, significant decreases in numbers of secondary and antral follicles, and decreased collagen in the ovarian stroma. *Plod1*, a lysyl hydroxylase that is vital for collagen crosslinking and deposition, was decreased at both the mRNA and protein levels in SIRT6‐deficient ovaries and granulosa cells (GCs). Additionally, we found abnormal estrogen levels in both SIRT6 KO mice and SIRT6 KD GCs, accompanied by decreases in the levels of the estrogen biosynthesis genes *Cyp11a1*, *Cyp19a1*, *Mgarp*, and increases in the levels of TNF‐α and NF‐κB. These results confirmed the effect of SIRT6 on follicular development and revealed a possible molecular mechanism for SIRT6 involvement in follicular development via effects on estrogen biosynthesis and collagen formation.

## Abstract 2

Our study confirmed that SIRT6 was involved in follicular development in mice in a way that SIRT6 deficiency downregulated the expression level of estrogen biosynthesis related genes *Cyp11a1*, *Cyp19a1*, *Mgarp*, and the expression level of collagen formation gene *Plod1*. And this molecular mechanism caused ovarian hypoplasia, which is characterized by disruptions of puberty and the estrus cycle, significant decreases in numbers of secondary and antral follicles, as well as decreased collagen in the ovarian stroma.

**Abbreviations**
AFs: atretic follicles
ChIP: Chromatin immunoprecipitation
CR: calorie restriction
GCs: granulosa cells
GFs: Graafian follicles
GO: Gene Ontology
KEGG: Kyoto Encyclopedia of Genes and Genomes
PmFs: primordial follicles
PrFs: primary follicles
SFs: secondary follicles; SIRT6 KD; SIRT6 knockdown
SIRT6 KO: SIRT6 knockout
SIRT6 OE: SIRT6 overexpression
WGCNA: weighted gene co‐expression analysis
WT: wild‐type

## 1 INTRODUCTION

Sirtuins are conserved nicotinamide adenine dinucleotide (NAD^+^)‐dependent deacetylases that have been reported to regulate lifespan in many organisms (Haigis & Sinclair, 2010; Lin et al., 2000). In mammalian genomes, there are seven members in the sirtuin family, SIRT1 to SIRT7 (Frye, 2000), of which SIRT6 has been shown to regulate metabolism, DNA damage repair and aging in mammals (Michishita et al., 2008; Mostoslavsky et al., 2006; Nakagawa & Guarente, 2014). SIRT6‐deficient mice are smaller in size and have a shortened lifespan; they usually develop abnormal premature aging phenotypes at 2–3 weeks and eventually die at approximately 4 weeks (Mostoslavsky et al., 2006). SIRT6 overexpression leads to a reduction in frailty and lifespan extension in both male and female B6 mice (Roichman et al., 2021).

To date, researchers have found that SIRT6 is essential in the development of many organs. SIRT6‐deficient mice developed abnormalities including cardiac hypertrophy (Sundaresan et al., 2012), profound lymphopenia (Mostoslavsky et al., 2006), decreased subcutaneous fat and osteopenia (Sugatani et al., 2015). However, whether SIRT6 is associated with gonad development is still poorly understood. The ovary is an important organ that belongs to the female reproductive system; it produces and releases oocytes periodically. Oocytes grow and develop within the environment provided by the ovarian follicles, which are composed of different numbers and types of cells according to the stage of folliculogenesis. The follicular development process can be divided into three stages: primordial follicles (PmFs), growing follicles, and Graafian follicles (GFs); according to size and structural differences, the growing follicles can be divided into primary follicles (PrFs) and secondary follicles (SFs). There are also some follicles that cannot be ovulated, which are called atretic follicles (AFs). Folliculogenesis ends when the ovaries are not capable of responding to the hormonal cues that recruit follicles to mature; this process signals the beginning of menopause.

Previous studies have shown that calorie restriction (CR) can prolong the female reproductive span, maintain the follicular reserve, and delay ovarian failure in adult rats (Liu et al., 2015), the effect were found to be associated with attenuation of mTOR signaling and increased level of ovarian SIRT1 and SIRT6 and other substrates FOXO3a and NRFI (Tatone et al., 2018). In rats, the ovarian damage induced by alkylating agents results in the decline of SIRT1, SIRT3, and SIRT6 expression and can be counteracted by Sirtuin activating strategies like resveratrol administration or CR (Said et al., 2016; Xiang et al., 2012) (Zhang et al., 2016). Serval researches have been conducted to see the role of SIRT6 in oocytes development. It has been proven that SIRT6 have a key role in controlling meiotic progression and its depletion after injection of SIRT6‐targeting morpholino resulted in disruption of spindle morphology and chromosome alignment in oocytes (Han et al., 2015). During the final oocyte growth phase, a significant increase in SIRT1 and SIRT6 transcripts was observed at the beginning of chromatin compaction in bovine oocytes (Labrecque et al., 2015; Tatone et al., 2018). These results indicated that SIRT6 plays an important role in the maintenance of ovarian follicles, yet the mechanism is still not clear enough; the effect of SIRT6 on other parts of ovaries such as granulosa cells (GCs) or stroma area remains unknown.

In this research, we first observed phenotypic differences in wild‐type (WT) and SIRT6 KO ovaries and found that SIRT6 KO ovaries developed abnormally. We also found that both the plasma estrogen level and the estrogen receptor expression level in the ovary were increased in SIRT6 KO mice. By conducting RNA‐seq analysis, a series of genes associated with hormone synthesis and metabolism were found to be impacted by the effects of SIRT6 on ovarian development. We also found decreases in *Plod1* gene and collagen expression in ovaries of SIRT6 KO mice, a lysyl hydroxylase that crosslinks collagen fibrils. These results confirmed the effect of SIRT6 on follicular development and revealed a possible molecular mechanism by which SIRT6 is involved in follicular development through effects on estrogen biosynthesis and collagen formation.

## 2 MATERIALS AND METHODS

### 2.1 Vaginal open time and estrus cycle

The vaginal open times of both WT and SIRT6 KO mice were observed and recorded. After the vagina had opened, to monitor the estrus cycle, vaginal smears were taken daily at 9:00–10:00 AM each day by using sterile cotton moistened with normal saline and then smeared on a clean glass slide (Long et al., 2018). The stage of the estrus cycle was determined based on vaginal cytology under a microscope as proestrus, estrus, metestrus or diestrus (Byers et al., 2012).

### 2.2 Pathological staining and immunohistochemical staining

Ovaries from WT and SIRT6 KO mice were fixed in 4% paraformaldehyde overnight at 4°C, dehydrated by ethanol and xylene and embedded in paraffin. Ovarian sections (5‐μm‐thick) were prepared by a motorized rotary microtome. The sections were next deparaffinized in xylene, hydrated through sequential ethanol washes and stained with HE and Masson's following standard protocols.

The immunohistochemical staining was conducted following the method described by SHI (Shi et al., 1991). For antigen retrieval, deparaffinized slices were placed in antigen retrieval buffer under microwave heating to temperatures up to 100°C. Endogenous peroxidase was blocked with 3% hydrogen peroxide buffer for 25 min. The tissue sections were then blocked with 3% BSA and stained with primary antibody (ERα or ERβ) and secondary antibody. Finally, the slices were stained with DAB buffer and hematoxylin for nuclear staining.

Both HE staining and immunohistochemical staining slices were scanned with an Axio Scan Z1 Slide Scanner (Zeiss), and pictures were captured by Zen Microscope and Imaging software at the same magnification.

### 2.3 Library construction, differential gene expression analysis and pathway analysis

The libraries were sequenced on the Illumina HiSeq 2000 platform, and the gene expression profiles of WT and SIRT6 KO ovaries were compared by using WGCNA to identify possible related signaling pathways and key regulatory genes. Interaction networks were produced to discover the central genes. For functional annotation, the DAVID Bioinformatics Resource was used. Then, we used KEGG, IPA, and GSEA for bioinformatics analysis.

### 2.4 SIRT6 knockdown (SIRT6 KD) and SIRT6 overexpression (SIRT6 OE) cell line construction and cell culture

The primary ovarian granulosa cell line from C57BL6 mice (BlueFBIO) was maintained in high‐glucose DMEM (HyClone) that contained 10% fetal bovine serum (Corning) and 1% penicillin/streptomycin (Corning). Cells were incubated at 37°C with 5% CO_2_ until reaching 80% confluence. Adherent cells were then treated with 0.25% trypsin (Corning) for 2 min, harvested, and expanded in T‐75 flasks (Corning). For SIRT6 KD cell line construction, lentivirus vectors encoding *Sirt6* shRNA were constructed, with the small interfering RNA (siRNA) duplexes targeting murine *Sirt6* as follows: sense: 5’‐GCCGTCTGGTCATTGTCAA‐3′, antisense: 5′‐TTGACAATGACCAGACGGC‐3′. The shRNAs were inserted into the GV314 lentiviral vector. For Sirt6‐overexpression cell line construction, lentivirus vectors encoding *Sirt6* cDNA were constructed (Shanghai Genechem Co., Ltd.). Recombinant viruses were packaged and amplified in 293 T cells and purified. The titer of viral particles was determined by the 50% tissue culture infective dose method, and then the viruses were transfected into GCs to produce *Sirt6* KD GCs.

### 2.5 *Cyp11a1*/*Cyp19a1*‐overexpression cell line construction, quantitative real‐time polymerase chain reaction (RT‐qPCR) and estradiol detection

The *Sirt6* KD ovarian granulosa cell line was maintained in high‐glucose DMEM (HyClone) that contained 10% fetal bovine serum (Corning), 1% penicillin/streptomycin (Corning) and 4 μg/mL puromycin. Cells were incubated at 37°C with 5% CO_2_ until reaching 80% confluence. Adherent cells were then treated with 0.25% trypsin (Corning) for 2 min, harvested, and expanded in T‐75 flasks (Corning). First, lentivirus vectors encoding *Cyp11a1/Cyp19a1* cDNA were constructed (LV‐Cyp11a1/Cyp19a1). The titer of viral particles was determined by the 50% tissue culture infective dose method, and then the viruses were transfected into GCs to produce *Cyp11a1/Cyp19a1*‐overexpression GCs. On the second day after lentivirus transfection, we screened with neomycin (200 μg/mL) and hygromycin B (300 μg/mL) respectively for 10 days to obtain *Cyp11a1/Cyp19a1*‐overexpression granulosa cell lines.

For RT‐qPCR analysis, total RNA was extracted from the above cells by TRIzol(Invitrogen). cDNA was obtained by using a Fast Quant RT Kit (Tiangen) and was subsequently used for RT‐qPCR analysis with SuperReal PreMix Plus (SYBR Green) (Tiangen) following the two‐step reaction program. The primers used for PCR analysis are shown in Table S1. Centrifuged cell culture supernates for 20 min at 1000 × ***g*** and collected the supernates and assay immediately. For estradiol detection, cell culture supernates was diluted following the guidelines provided by the ELISA kit (Cloud‐Clone Corp).

### 2.6 Chromatin immunoprecipitation(ChIP) assay

ChIP assay was performed using ChIP‐IT Express Enzymatic Magnetic Chromatin Immunoprecipitation Kit (Active Motif) following the manufacturer's protocols. WT, SIRT6 KD, and SIRT6 overexpression (OE) ovarian granulosa cell lines were used as materials and special primers were designed to amplify the promoter sequence of *Cyp11a1*, *Plod1*, and *Mgarp*. The primers used for PCR analysis are shown in Table S2. The antibodies specific for SIRT6 were purchased from Abcam, and the control antibody of normal rabbit IgG was purchased from Cell Signaling Technology.

### 2.7 Statistical analysis

The data were evaluated with GraphPad Prism software, and the results are shown as the mean ± SEM of at least three independent experiments. The *p* values of comparisons between WT and SIRT6 KO mice were calculated by two‐tailed Student's *t* test. For multigroup comparisons, one‐way ANOVA was used. For multigroup comparisons with two variables, two‐way ANOVA was used. *p < 0.05* was considered to indicate that the differences were statistically significant.

## 3 RESULTS

### 3.1 Vaginal opening and estrus cycle were not observed in SIRT6 KO mice

**FIGURE 1:**
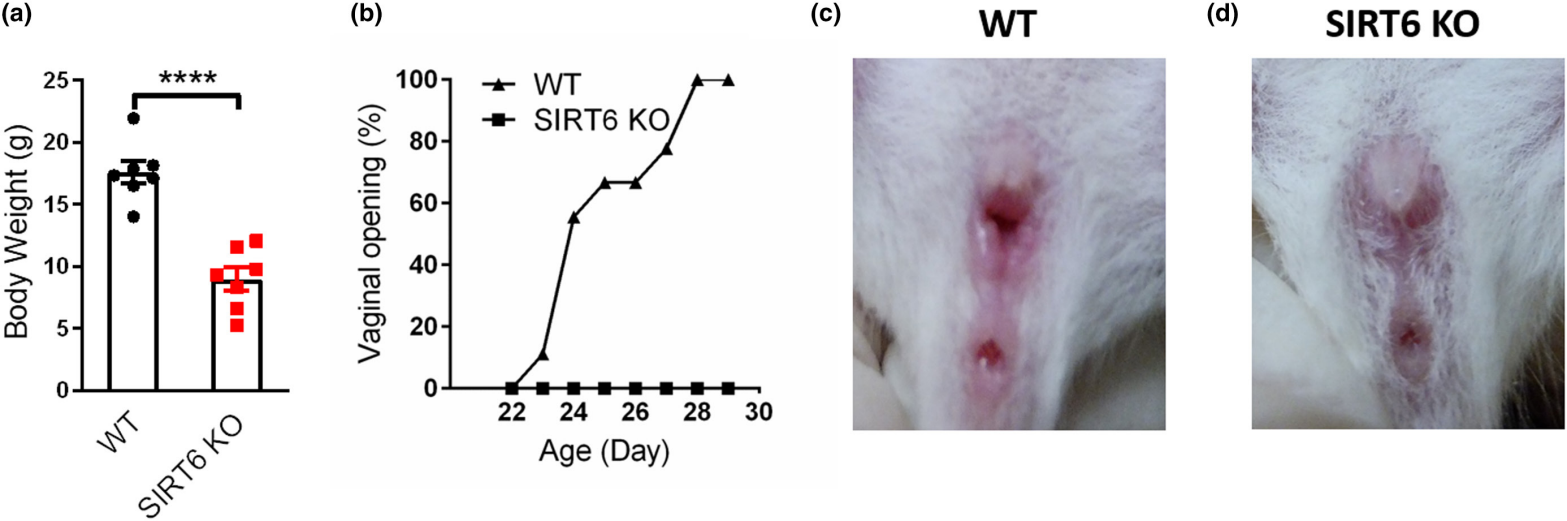
(a) Body weight of 4‐week‐old SIRT6 KO mice compared with their WT littermates mice (female). (b) Vaginal opening rate of WT and SIRT6 KO mice. (C‐D) Representative vaginal orifice images of WT (B) and SIRT6 KO (c) mice from the same litter on the 28th day after birth. (*n* = 7 for each genotype). ****p < 0.0001.

Previous studies have shown that knockout of the *Sirt6* gene leads to a dramatically shortened life span, smaller size, and a premature aging‐like phenotype (Mostoslavsky et al., 2006). In our research, both female and male SIRT6 KO mice showed a significantly smaller size and decreased weight compared with WT mice (Figure 1a). Both vaginal opening time and estrus cycle were observed and recorded according to previous reported methods (Byers et al., 2012; Long et al., 2018) (Figure 1b). The vaginal orifice of SIRT6 KO mice did not open until they died at an average of 29.25 days, while the average vaginal opening age of their WT littermates was 24.67 days (Figure 1c,d; Table 1). As a result, SIRT6 KO mice did not show a regular estrus cycle, indicating abnormal development of the female reproductive system.

**TABLE. 1:** The average vaginal opening day and estrus cycle of WT and SIRT6 KO mice.

	WT	SIRT6 KO
Vaginal opening (day)	24.67 ± 0.56	Do not open until die (average lifespan 29.25 ± 1.32*)
Estrus cycle (day)	5.29 ± 0.36	Do not have estrus cycle

* *p* = 0.0323

### 3.2 Abnormal development of ovarian follicles in SIRT6 KO mice

**FIGURE 2:**
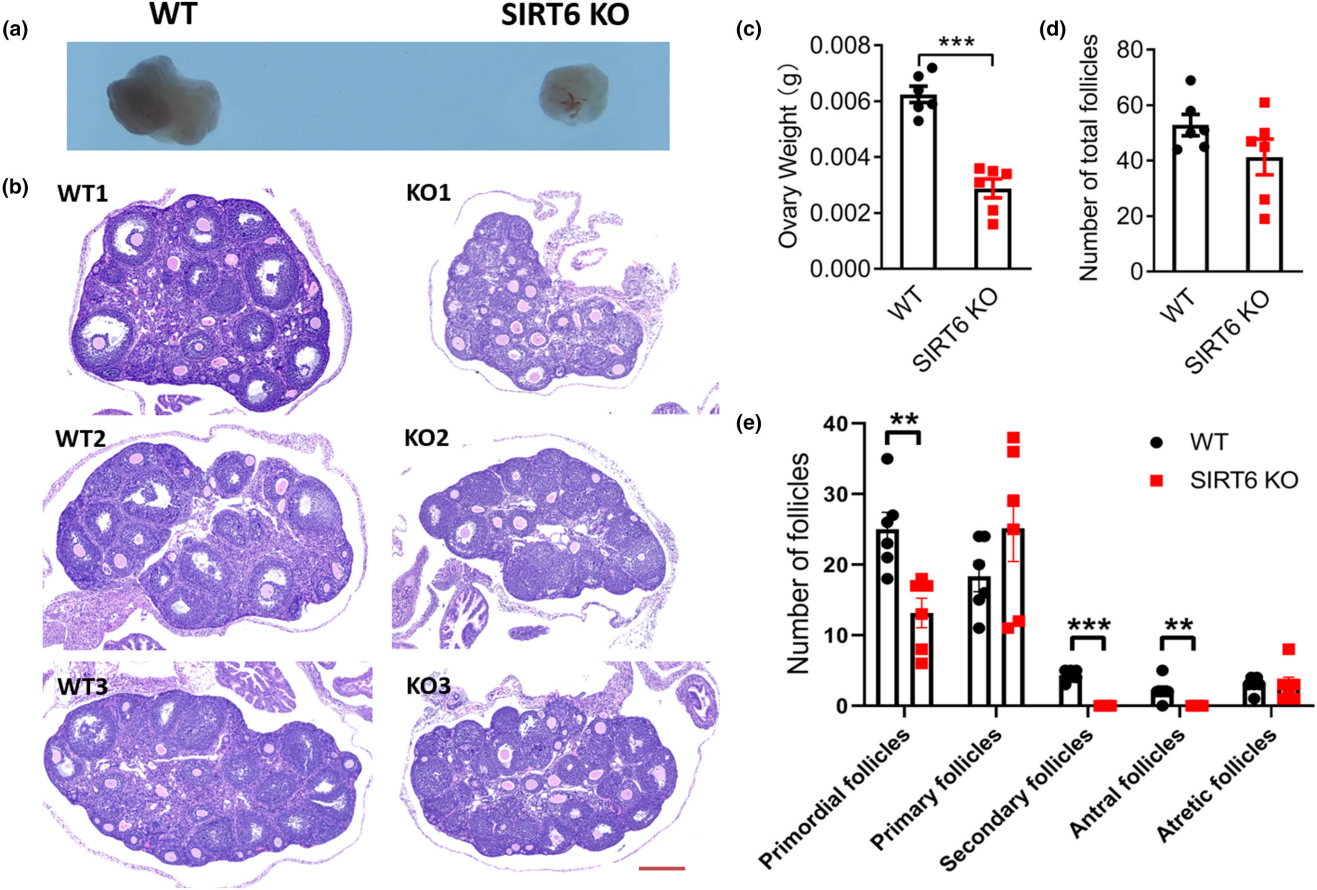
(a) Size differences and (b) histological analysis of ovaries from 4‐week‐old WT and SIRT6 KO mice. (c) Ovary weight of WT and SIRT6 KO mice. (d) Total numbers of ovarian follicles. (e) Number of follicles at different stages; secondary and antral follicle numbers were significantly decreased in SIRT6 KO mice compared with WT mice (*n* = 6 for each genotype). *p < 0.05, **p < 0.01, ***p < 0.001. Scale bars: 200 μm.

The ovaries from WT and SIRT6 KO mice were collected, proteins of the mouse right ovaries were extracted and then the expression of SIRT6 was verified by western blot (Figure S1A). The onset of puberty and estrus cycle were closely linked with follicle development and plasma estrogen level, therefore, we observed the histomorphological differences of ovaries from WT and SIRT6 KO mice. The results showed that ovaries from SIRT6 KO mice were much smaller in size than the WT ovaries (Figure 2a,c). Fewer stromal areas were found in SIRT6 KO ovaries (Figure 2b). The total ovarian follicles of WT and SIRT6 KO mice showed no significant differences (Figure 2b,d). However, most of the ovarian follicles in SIRT6 KO ovaries were immature and those cells in ovarian follicles were less differentiated and had an irregular arrangement (Figure 2b,e). Primordial follicles in SIRT6 KO ovaries were significantly decreased, indicating a weakened ovarian reserve capacity (Figure 2b,e). Furthermore, no SFs or antral follicles were found in SIRT6 KO ovaries (Figure 2e), indicating that the follicle development were blocked at primary follicle stage in SIRT6 KO ovaries, which was consistent with the delayed puberty. The histomorphological analysis of both WT and SIRT6 KO ovaries indicated that the lack of SIRT6 caused ovarian hypoplasia.

### 3.3 Estrogen biosynthesis related genes *Cyp11a1* and *Cyp19a1* were downregulated in ovaries of SIRT6 KO mice

**FIGURE 3:**
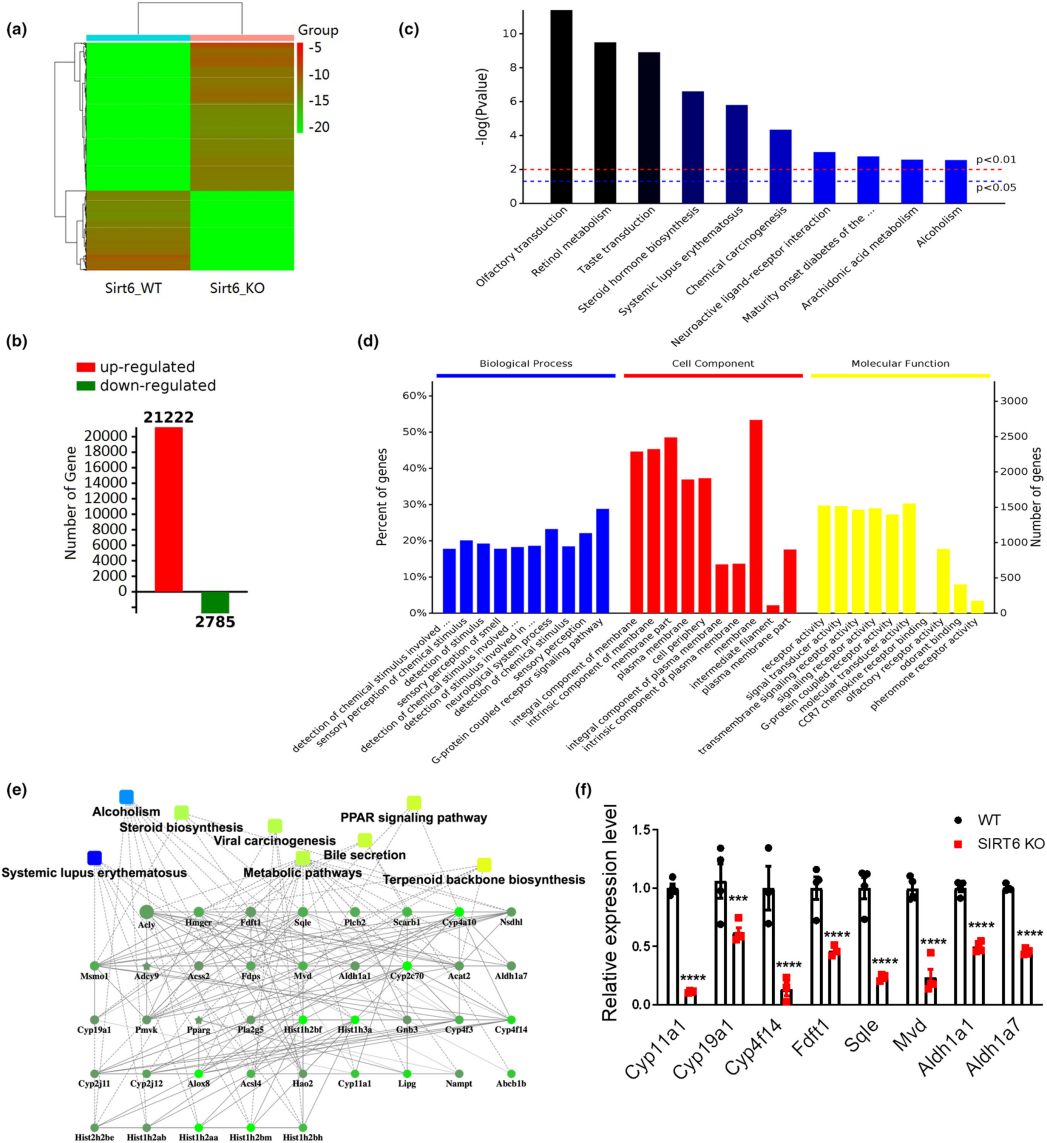
(a) Heatmap depicting the genes that are up‐ and downregulated in SIRT6 KO ovaries compared with WT ovaries. (b) Statistical graph showing the number of up‐ or downregulated genes in SIRT6 KO ovaries compared with WT ovaries. Red: upregulated genes; green: downregulated genes. (d) Gene Ontology (GO) and (c) Kyoto Encyclopedia of Genes and Genomes (KEGG) enrichment analysis. (e) Key hub genes and associated functional annotations identified from the network analysis. (f) RT‐qPCR verification of downregulated genes discovered by RNA‐seq analysis. *n* = 4, ***p < 0.001, ****p < 0.0001.

To further explore the underlying mechanism of ovarian hypoplasia caused by SIRT6 deficiency, RNA‐seq was conducted to find out the differentially expressed genes between WT and SIRT6 KO ovaries. The heatmap shows the reads per kilobase of exon model per million mapped reads (RPKM) value in green (downregulated) or red (upregulated), depending on which we found 21,222 activated genes and 2758 reduced genes in the SIRT6 KO ovaries compared with the WT ovaries (Figure 3a, b). Differentially expressed genes were then subjected to GO (Gene Ontology) and KEGG (Kyoto Encyclopedia of Genes and Genomes) enrichment analysis. Thirty biofunction items through GO analysis (Figure 3d) and 10 pathways from KEGG analysis (Figure 3c) that were significantly changed in SIRT6 KO ovaries were listed. GO analysis showed that the differentially expressed genes were mostly classified as cellular component like membrane part and integral component of membrane (Figure 3d). KEGG analysis indicated 10 significant pathways, including olfactory transduction, retinol metabolism, taste transduction, and steroid hormone biosynthesis (Figure 3c). Combined with ovary function, we are mostly interested in genes that are related with steroid hormone biosynthesis.

To better understand the connections between the differentially expressed genes and pathways that are closely related with steroid hormone biosynthesis, we conducted weighted gene co‐expression analysis (WGCNA). And the analysis of the gene network from WGCNA includes the quantification of gene connections (Figure 3e). Several key hub genes involved in cholesterol or steroids biosynthesis were picked out and verified by RT‐qPCR, and all of them were significantly downregulated (Figure 3f). *Cyp11a1*, *Cyp19a1*, and *Cyp4f14* belong to the cytochrome P450 superfamily, and they catalyze many reactions involved in synthesis of cholesterol, steroids and other lipids. FDFT1, SQLE, and MVD are three enzymes involved in different stages of cholesterol biosynthesis. *Aldh1a1* and *Aldh1a7* are two genes belonging to the aldehyde dehydrogenase family, and they are also involved in cholesterol synthesis.

Of all the detected genes, *Cyp11a1* and *Cyp19a1* are closely linked with estrogen biosynthesis (Tsuchiya et al., 2005). *Cyp19a1* is commonly used as a marker gene for GCs, and it is up regulated in large antral follicles (>9 mm) compared with much smaller follicles (3–5 mm) (Bao & Garverick, 1998; Hatzirodos et al., 2015). The ovarian follicles in SIRT6 KO mice were mostly blocked to PrFs (smaller follicles) and do not have secondary or antral follicles (large follicles) (Figure 2b,e). And the expression of *Cyp19a1* in ovaries of SIRT6 KO mice was also significantly downregulated (Figure 3f), which is consistent with the previous study (Hatzirodos et al., 2015). Another study conducted by Miaomiao Wang et al found that the stable expression of CYP11A1 was crucial for the normal states and functions of follicles, and the expression level of *Cyp11a1* was significantly reduced in growth‐impaired follicles compared with that in healthy follicles (Wang, Wang, et al., 2022). These results indicate that the ovarian hypoplasia appearance in SIRT6 KO mice may be related with the unstable expression of *Cyp11a1* and *Cyp19a1*.

### 3.4 SIRT6 knockdown inhibited the function of GCs to synthesis estradiol

**FIGURE 4:**
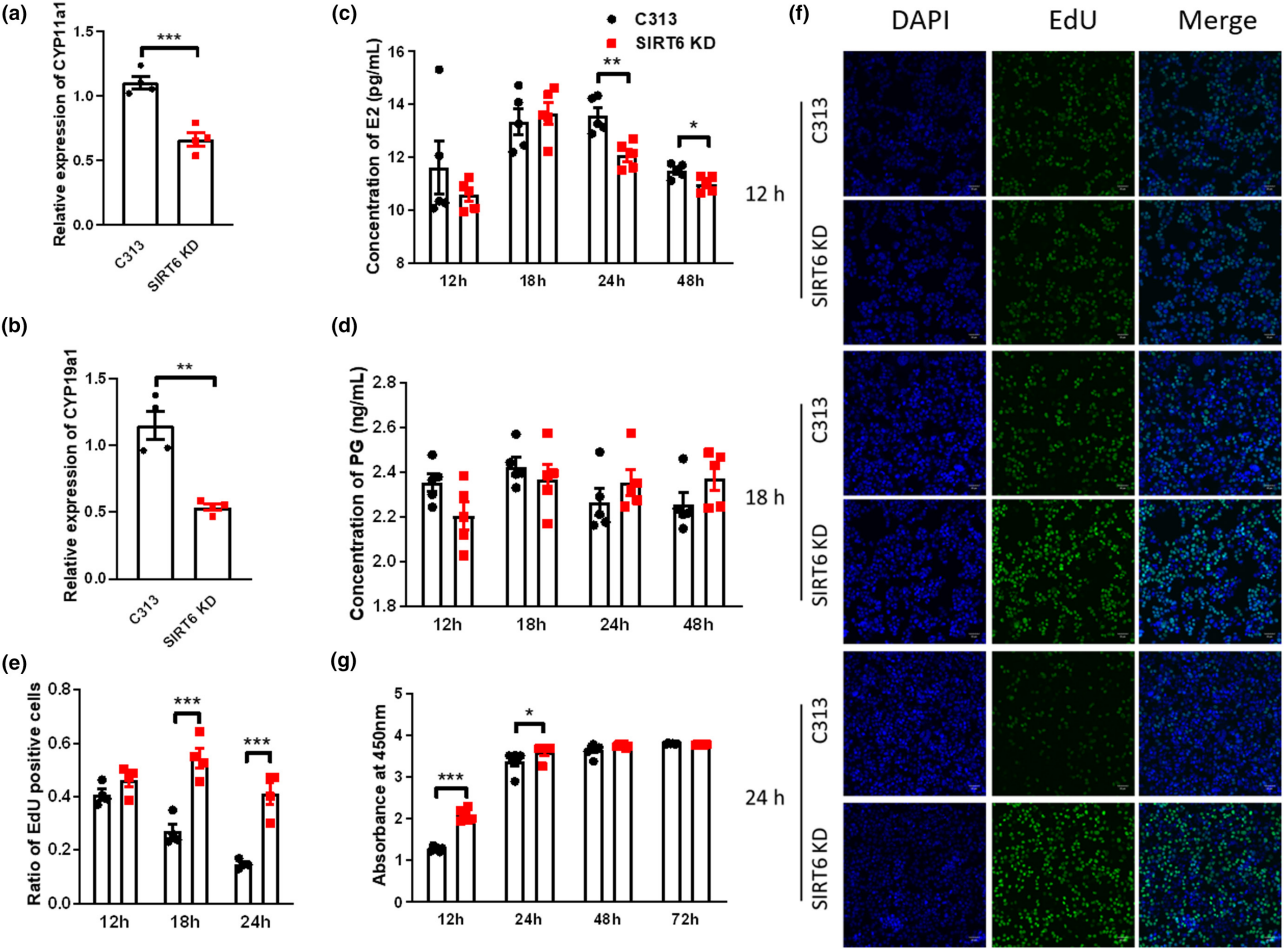
*Sirt6* knockdown inhibited the function of granulosa cells (GCs) to synthesize estrogen and led to the excessive proliferation of GCs. (a, b) RT‐qPCR results of *Cyp11a1* and *Cyp19a1* in GCs cultured for 24 h (*n* = 4). (c, d) Knockdown of SIRT6 in GCs decreased the secretion of estradiol (c), but not the PG level (d), after culture for 24 and 48 h (*n* = 5). (e) A CCK‐8 assay was conducted to detect granulosa cell (GC) viability in cells transfected with lenti‐control (C313) and lenti‐si*Sirt6* (SIRT6 KD) viruses (*n* = 4). (f, g) The cell division rate was increased in the SIRT6 KD group (*n* = 4). *p < 0.05; **p < 0.01; ***p < 0.001.

The ovary possesses of three types of cells: oocytes, GCs, and theca cells. The theca cells are responsible for androgen synthesis, and GCs are responsible for conversion of androgens to estrogens, as well as progesterone synthesis (Havelock et al., 2004). To further investigate the mechanism of SIRT6 in estrogen synthesis, we established a *Sirt6* knockdown follicle granulosa cell model by transfection with lentivirus containing the *Sirt6* siRNA sequence. The cell line model was verified by RT‐qPCR, and the mRNA level of *Sirt6* was decreased as predicted (Figure S1B). *Cyp11a1* and *Cyp19a1* genes were also significantly decreased after *Sirt6* knockdown (Figure 4a,b), which is consistent with our observation in SIRT6 KO mice (Figure 3f). In the cellular experiments, the estradiol and progesterone (PG) levels in the supernatant of cultured SIRT6 KD follicle GCs were measured. The level of estradiol in SIRT6 KD follicle GCs was found to be significantly decreased compared with that in the control group at 24 and 48 h (Figure 4c), but the level of PG did not show a large difference (Figure 4d).

Both EdU and CCK‐8 assays were conducted to evaluate the effect of SIRT6 deficiency on cell proliferation of follicle GCs. The results showed that the knockdown of the *Sirt6* gene led to the excessive proliferation of GCs during the first 24 h of cultivation (Figure 4e–g), and both cell viability and the number of newly proliferated cells were significantly increased (Figure 4e–g). Previous study conducted by Zhang et al showed that SIRT6 expression is significantly reduced in human ovarian cancer tissues compared to normal tissues. Downregulation of SIRT6 enhanced the proliferation of ovarian cancer cells, while SIRT6 OE inhibited their growth (Zhang et al., 2015). Another study showed that knockdown of SIRT6 significantly promoted clone formation ability in the HuH7 cell (human liver cancer cells), and SIRT6 overexpression inhibited cell clone formation through inhibition of the ERK1/2 signaling pathway (Wang, Lan, et al., 2022). Investigation of hepatocellular carcinoma demonstrated that SIRT6 suppressed the NF‐κB activation, and markedly impaired the initiation and development of cancer cells (Min et al., 2012).

Next, plasma from both WT and SIRT6 KO mice was extracted and analyzed by ELISA. However, we found that SIRT6 KO mice showed increased estrogen (E) and estradiol (E2) levels (Figure S2A,B) and unchanged follicle‐stimulating hormone (FSH) level (Figure S2E). According to clinical treatment guideline, the increase in basal estradiol level often suggests the reduction of ovarian reserve function, while normal FSH with an increase in estradiol level usually represents the early stage of the reduction (Medicine, 2019). Gonadotropic hormone (GTH), which mainly includes luteinizing hormone (LH) and FSH, was significantly decreased in SIRT6 KO mice (Figure S2C), mainly due to the decrease in LH (Figure S2D). The level of gonadotrophin releasing hormone (GnRH) did not show much difference (Figure S2F). We also found that inflammatory factors including ac‐NF‐κB, NF‐κB, and TNF‐α were increased in SIRT6 KO mice (Figure S2G–J).

### 3.5 SIRT6 KO leads to decreased expression of the *Plod1* gene and collagen expression in SIRT6 KO mice

**FIGURE 5:**
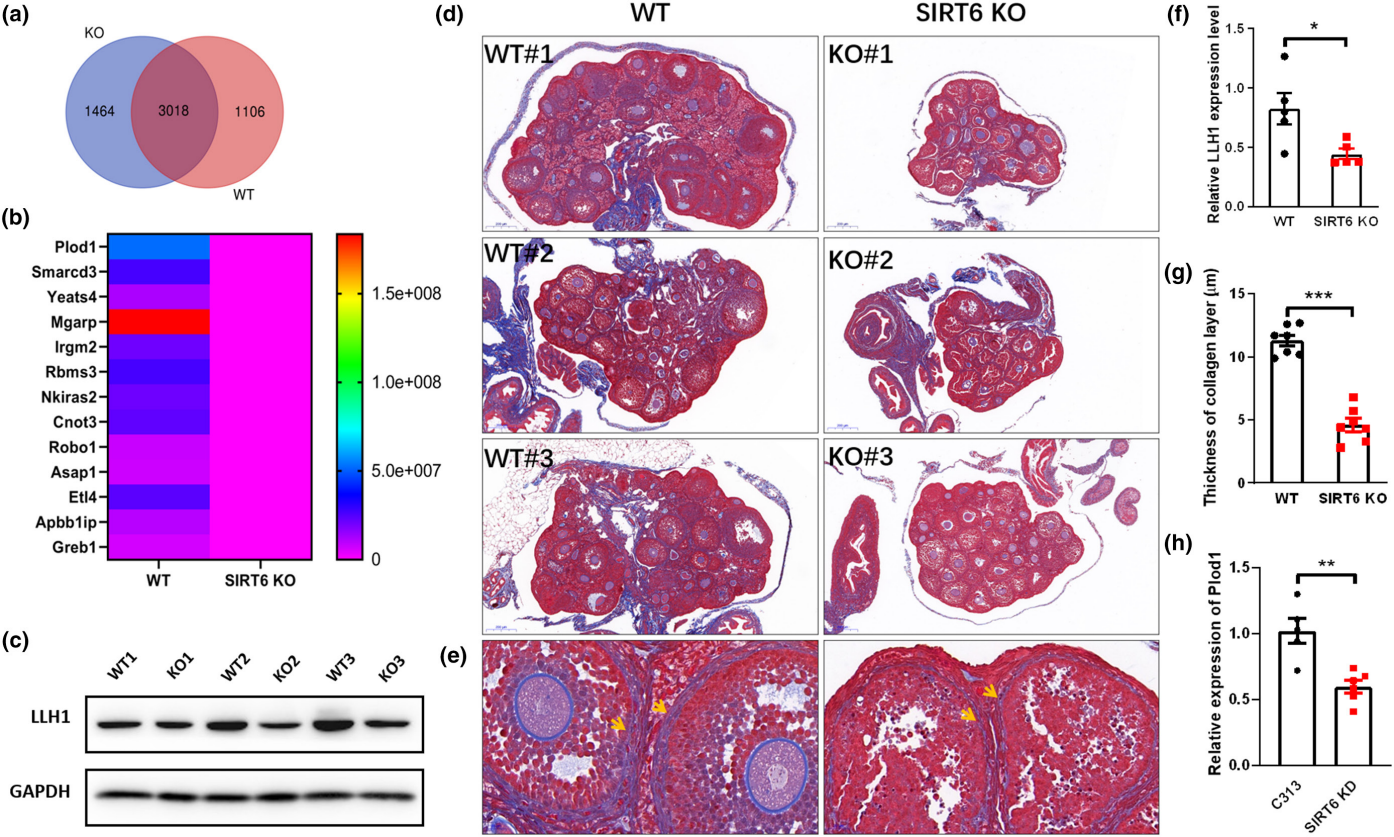
SIRT6 KO led to decreased expression of the *Plod1* gene. (a) Venn diagram representing protein expression differences in WT and SIRT6 KO ovaries. (b) Thirteen proteins were not expressed in SIRT6 KO ovaries, according to proteomic analysis. (c) Western blot of LLH1 in ovaries from WT and SIRT6 KO mice. (d) Masson staining of ovaries of WT and SIRT6 KO mice showing the collagen content, each group showed three pictures. Scale bars: 200 μm. (e) The partial enlarged view of WT#1 and KO#1. Scale bars: 20 μm. (f) Quantification analysis of western blot result of LLH1 (*n* = 5). (g) Quantification analysis of collagen layer thickness in WT and SIRT6 KO ovaries. (*n* = 7). (h) Relative expression of *Plod1* in SIRT6 KD cell line (*n* = 5). *p < 0.05, ** *p* < 0.01, *** *p* < 0.001.

Since the plasma level of estrogen in SIRT6 KO mice is adequate to guarantee the development of follicles (Figure S2A,B), it is highly possible that the arrest of follicular development was caused by other factors. To find out the molecular mechanism of SIRT6 in follicular development, proteomics sequencing was conducted, and we found a special series of proteins that were silenced in SIRT6 KO mice (Figure 5a,b), of which the expression of Mitochondria‐localized glutamic acid‐rich protein (MGARP) changed the most (Figure 5b). MGARP is a novel mitochondrial transmembrane protein expressed mainly in steroidogenic tissues. Previous studies showed that MGARP functioned in hormone biosynthesis and its expression was modulated by the HPG axis (Zhou et al., 2011), and it was also identified as a new potential marker for GCs. The expression of *Mgarp* was upregulated in large follicles (>9 mm) compared with small follicles (3–5 mm) (Long et al., 2018), the expression profile change was similar as *Cyp19a1* and was consistent with what we observed in SIRT6 KO ovaries (smaller follicles) (Figure 3f).

PLOD1 is a secondly changed silenced protein (Figure 5b), and it is a lysyl hydroxylase that is vital for collagen cross‐linking and deposition, as well as for LLH1 biosynthesis (Qi & Xu, 2018). Lysyl hydroxylase is a membrane‐bound homodimeric protein localized to the cisternae of the endoplasmic reticulum that catalyzes the hydroxylation of lysyl residues in collagen‐like peptides. The resultant hydroxylysyl groups are attachment sites for carbohydrates in collagen, and thus, they are critical for the stability of intermolecular crosslinks (Takaluoma et al., 2007). The results showed that the expression levels of *Plod1* and its related protein LLH1 were significantly decreased in the ovaries of SIRT6 KO mice (Figure 5b,c,f). Masson staining showed that the collagen thickness in SIRT6 KO ovaries was significantly decreased (Figure 5d,e,g). The expression of *Plod1* in SIRT6 KD GCs was also downregulated (Figure 5h).

### 3.6 Restoring *Cyp11a1* and *Cyp19a1* in SIRT6 KD cells could rescue the expression level of *Plod1* and estrogen level

**FIGURE 6:**
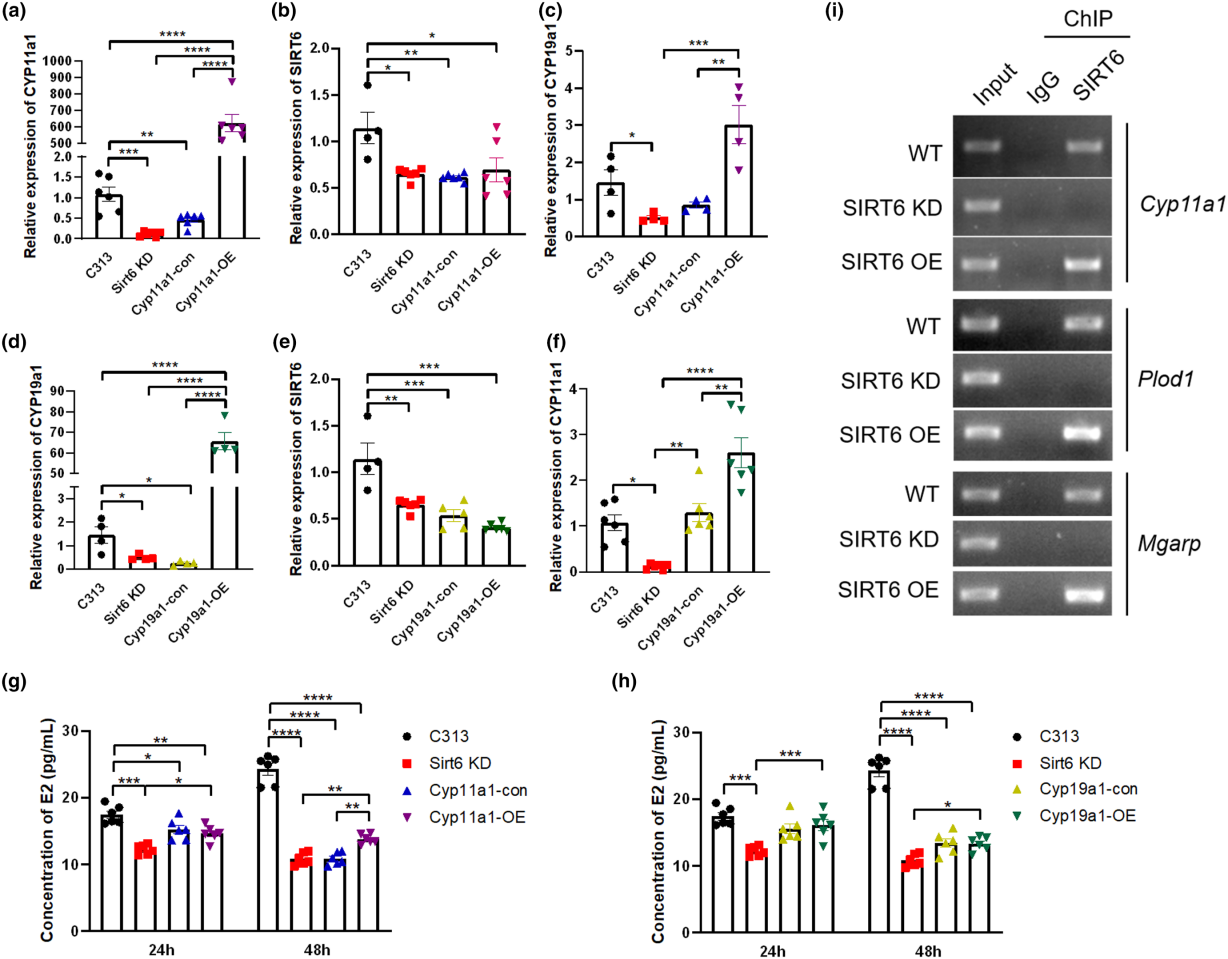
Restoring *Cyp11a1* or *Cyp19a1* genes in SIRT6 KD cells can rescue the estrogen level. (a–c) Relative expression of *Cyp11a1, Sirt6, Cyp19a1* in *Cyp11a1*‐OE SIRT6 KD cell line. (d–f) Relative expression of *Cyp11a1, Sirt6, Cyp19a1* in *Cyp19a1*‐OE SIRT6 KD cell line. (g, h) Concentration of estradiol in the cell culture medium in different cell types. (i) ChIP‐seq results showed that SIRT6 bound to *Cyp11a1*, *Plod1* and *Mgarp* promoters. *p < 0.05, **p < 0.01, *** *p* < 0.001, **** *p* < 0.0001 (*n* = 4–6 for each group).

Both *Cyp11a1* and *Cyp19a1* overexpression cell lines were built based on SIRT6 KD cell line to see whether restoring the two genes in SIRT6 KD cells could rescue the decreased estradiol level (Figure 6a,d). The overexpression of *Cyp11a1* and *Cyp19a1* in SIRT6 KD cells did not cause the change of *Sirt6* expression (Figure 6b,e). The overexpression of *Cyp11a1* can compensate the downregulation of *Cyp19a1* caused by *Sirt6* knockdown while *Cyp19a1* compensates the downregulation of *Cyp11a1* level caused by *Sirt6* knockdown (Figure 6c,f). The decreased estradiol level caused by *Sirt6* knockdown were also restored partly by *Cyp11a1* and *Cyp19a1 overexpression* (Figure 6g,h), indicating that *Cyp11a1* and *Cyp19a1* are two important downstream genes regulated by SIRT6 in estrogen synthesis and metabolism.

### 3.7 SIRT6 directly binds to the promoter of *Plod1*, *Mgarp* and *Cyp11a1* gene and regulates their transcription

**FIGURE 7:**
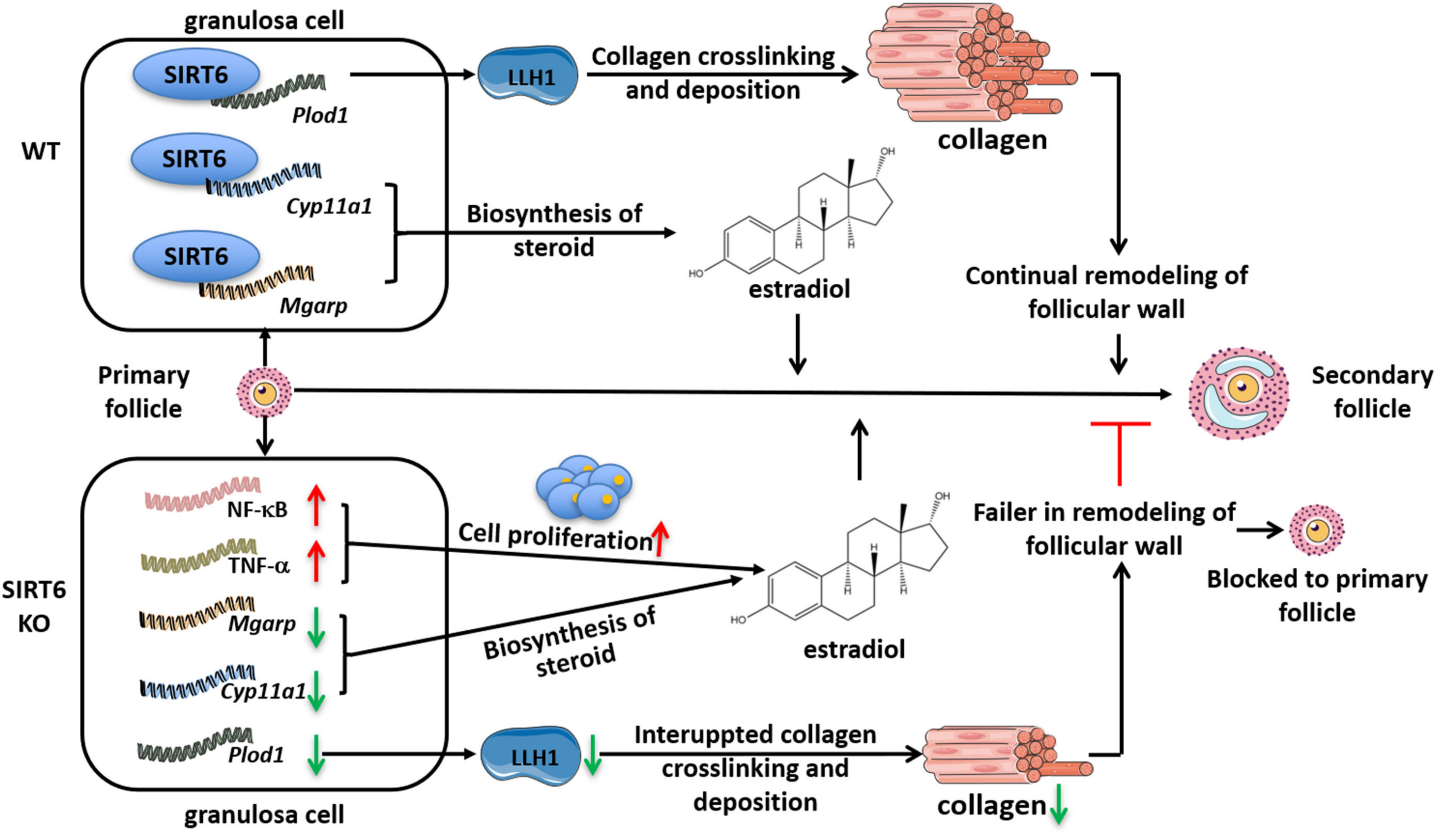
Schematic diagram of the molecular mechanism of SIRT6 in regulating follicle development.

Previous ChIP‐seq resulted from Cistrome Data Browser showed that SIRT6 bound to *Plod1, Mgarp*, *and Cyp11a1* promoters in mouse embryo tissue (Etchegaray et al., 2015) (Figure S3). To confirm whether a similar mechanism occurs in mouse follicle GCs, ChIP assay was conducted to further clarify the interaction between SIRT6 and the three genes. Several pairs of primers around predicted binding site of *Plod1, Mgarp*, and *Cyp11a1* genes were designed. We found increased binding of SIRT6 at the promoters of *Plod1, Mgarp*, and *Cyp11a1* in SIRT6 OE cell line compared with the WT mouse follicle GCs, whereas almost no binding was detected in SIRT6 KD follicle granulosa cell line. These results suggest that SIRT6 regulates the expression of *Plod1, Mgarp* and *Cyp11a1* by directly binding to their promoter regions (Figure 6i). A schematic diagram was made to show the possible molecular mechanisms of SIRT6 in regulating follicle development (Figure 7).

## 4 DISCUSSION

In this research, we explored the effect of SIRT6 on ovary development and found that SIRT6 deficiency leads to ovarian hypoplasia, disruptions of puberty, and the estrus cycle. Primordial follicles were significantly decreased, most of the follicles were blocked at primary follicle stage with no secondary or antral follicles in SIRT6 KO mice (Figure 2).

Previous study showed that GCs from small and large follicles differed substantially in their transcriptomes (Hatzirodos et al., 2015; Taira & Beck, 2007). *Cyp19a1 and Mgarp* are commonly used as marker genes for GCs (Long et al., 2018), and they were both upregulated in large antral follicles (>9 mm) compared with much smaller antral follicles (3–5 mm) (Long et al., 2018). *Cyp11a1* is another steroidogenic enzyme participant in estrogen synthesis. In human ovaries, exogenous toxin‐mediated downregulation of *Cyp11a1* gene expression level leads to disruption of estradiol synthesis, reduced ovulation rate and infertility (Wang, Wang, et al., 2022). In mouse ovaries, the expression level of *Cyp11a1* was significantly reduced in growth‐impaired follicles compared with that in healthy follicles (Wang, Wang, et al., 2022). We found that the expression of *Cyp19a1, Mgarp*, and *Cyp11a1* were decreased in SIRT6 deficiency ovaries and granulosa cells (Figures 3, 4, 5), which is consistent with the impaired follicular development in SIRT6 KO mice. The secretion of estradiol was also downregulated in SIRT6 KD granulosa cells, which were partly recovered after overexpression of *Cyp11a1* or *Cyp19a1* (Figure 6), indicating that SIRT6 deficiency weakened hormone synthesis ability of granulosa cells, and SIRT6 regulates the expression of *Mgarp* and *Cyp11a1* by directly binding to the promoter areas of the two genes (Figure 6).

Our results showed that the estradiol levels secreted by SIRT6 KD granulosa cells were decreased, but the estrogen and estradiol levels in the plasma of SIRT6 KO mice were significantly increased (Figure S2). A reasonable explanation for this contradictory result is that even though SIRT6 weakened hormone synthesis ability of granulosa cells, it also accelerated the proliferation rate of granulosa cells. When the amount of cells was similar (such as in vitro condition and cultured in dishes), the ability of granulosa cells to secrete estrogen was weak in SIRT6 deficiency group. Once SIRT6 deficiency group have more granulosa cells and the higher cell number would counteract their functional defect, the total estrogen or estradiol level secreted by granulosa cells would be higher in SIRT6 deficiency group. According to clinical treatment guideline, normal FSH level with an increase in estradiol level in plasma usually represents the early stage of the reduction (Medicine, 2019), which is consistent with what we have observed in SIRT6 KO mice (Figure S2A,B,E). The follicles in SIRT6 KO ovaries were mostly blocked to primary follicle (Figure 2), it is hard to calculate whether there are more granulosa cells in SIRT6 KO ovaries than in WT ovaries. More research work is needed to further explain this phenomenon.

Previous study showed that SIRT6 suppressed the NF‐κB activation, and markedly impairs the initiation and development of cancer cells (Min et al., 2012). SIRT6 expression is significantly reduced in human ovarian cancer tissues compared to normal tissues, and downregulation of SIRT6 enhanced the proliferation of ovarian cancer cells. In accordance with previous study (Kawahara et al., 2009), we found that the inflammatory factors including ac‐NF‐κB, NF‐κB, and TNF‐α were increased in SIRT6 KO mice (Figure S2), which are important proteins that promotes cancer cell proliferation. Further exploration is needed to figure out whether the accelerated cell proliferation of SIRT6 KD granulosa cells shares the same mechanism as in cancer cells.

Collagens, a large family of glycoproteins, are the structural building blocks of tissues and are the major component of extracellular matrix (ECM). ECM is extremely important for the follicular development, it helps follicular fluid formation, filters soluble materials, and provides rigid or elastic mechanical support for tissues. Previous study showed that the lack of focimatrix can inhibit the depolarization of the membrana granulosa transformation (Irving‐Rodgers & Rodgers, 2006; Saha et al., 2005), leading to the retardation of follicular development.

*Plod1* is a lysyl hydroxylase that is vital for collagen crosslinking and deposition. Previous study showed that hypothyroid condition led to reproductive disorders in female rats, it functioned mainly by disturbing collagen biosynthesis in ovary through downregulating the expression of key enzymes (*Plod1*, *Plod2*, and *Plod3*) for the first step of collagen biosynthetic pathway. During follicular development, continual remodeling of the follicular wall occurs as it enlarges, downregulation of Plod causes inhibition of new collagen formation or even the overall disturbance of collagen status and also the ovarian structure, leading to the improper follicular development, which may also affect the steroidogenesis process.

In this study, most follicles in SIRT6 KO mice were stagnated in PrFs, we also observed decreased collagen expression in the ovarian stroma in SIRT6 KO mice, combined with downregulated *Plod1* level at both the mRNA and protein levels in SIRT6‐deficient granulosa cells and ovaries (Figure 5). The ChIP assay has demonstrated that SIRT6 bound directly with *Plod1* gene and regulate its expression (Figure 6). Our results indicated that SIRT6 deficiency caused improper follicular development through downregulating the *Plod1* expression and collagen formation.

In conclusion, our study discovered for the first time that SIRT6 deficiency inhibited follicular development, which led to the delayed puberty and estrus cycle. Our results confirmed the effect of SIRT6 on follicular development and revealed that SIRT6 involved in follicular development via affecting *Plod1* and its related collagen formation. This research helps us to have a deeper understanding of ovarian development process, and provides a theoretical basis for the causes of ovarian dysfunction related diseases, which will help us to discover new interventions to improve the health status of ovaries.

## AUTHOR CONTRIBUTIONS

Liyuan Li, Zhao Wang, Kaiqiang Hu, and Shangfeng Liu designed the experiments. Liyuan Li, Rui Hua, Kaiqiang Hu, Huiling Chen, and Yuemiao Yin performed the animal experiments. Rui Hua, Xiaojin Shi, Kezheng Peng, and Qing Huang performed the cellular experiments. Liyuan Li, Rui Hua, Ying Qiu, and Xue Li analyzed the data. Liyuan Li, Qingfei Liu, and Zhao Wang wrote the manuscript.

## CONFLICT OF INTEREST STATEMENT

The authors declare no conflict of interest.

## Supporting information


Data S1.

## Data Availability

The data that support the findings of this study are available from the corresponding author upon reasonable request.
