# Supplementary material for: SIRT6 deficiency causes ovarian hypoplasia by affecting Plod1‐related collagen formation
Source: Aging Cell. 2023 Nov 7;23(2):e14031. doi: 10.1111/acel.14031 (PMC10861214; doi:10.1111/acel.14031)
Supplement: Supplementary file 1 — Data S1. [file ACEL-23-e14031-s001.docx]

Supplementary Data

SIRT6 deficiency causes ovarian hypoplasia by affecting *Plod1* related collagen formation

Liyuan Li^1,2#^, Rui Hua^1#^, Kaiqiang Hu^1^, Huiling Chen^1^, Yuemiao Yin^1^, Xiaojin Shi^1^, Kezheng Peng^1^, Qing Huang^1^, Ying Qiu^3^, Xue Li^3^, Qingfei Liu^1^, Shangfeng Liu^4^, Zhao Wang^1,*^

**Liyuan Li^#^ and Rui Hua^#^ contributes equally to this article.**

**^Methods:^**

***Animals and housing conditions***

*Sirt6^tm1.1Cxd^* mice (purchased from Jackson Laboratory, USA) were crossed with CMV-cre and maintained by the Nanjing Biomedical Research Institute. 129Sv SIRT6^+/-^ male mice were crossed with C57BL6 female mice for several generations to obtain 129Sv/C57BL6 SIRT6^+/-^ mice. Both WT (genotype SIRT6^+/+^) and Sirt6 KO (genotype SIRT6^-/-^) female mice (4 weeks old) were used for research. All animals were housed in cages in the laboratory animal center of Tsinghua University with free access to food and water in a room with an ambient temperature of 22°C ± 2°C and a 12-hour light:12-hour dark cycle. All mouse experiments were conducted according to the relevant guidelines and regulations and with the approval of the Institutional Ethical Committee of China. Tail tips from 2-week-old mice were obtained to extract genomic DNA for genotyping analysis, and the following primers were used: Fw: 5’-AGTGAGGGGCTAATGGGAAC-3’;Rv1:5’-CTGACGGTGTCTTCACAAACTCAC-3’; Rv2: 5’-AACCCACCTCTCTCCCCTAA -3’. Animals were obtained as described above, and all the mice used in the research were evaluated by genotyping and Western blotting to detect SIRT6 expression.

***Plasma hormone detection and analysis***

Four-week-old animals were anesthetized by intraperitoneal injection of avertin, and blood samples were obtained and collected into anticoagulation tubes by cardiac puncture. Plasma was separated by centrifugation at 1000 rpm for 5 min. For hormone detection, plasma was diluted following the guidelines provided by the ELISA kit. The ELISA kit information was as follows: luteinizing hormone (LH) (Cloud-Clone Corp, Houston, USA); estrogen (E), estradiol (E2), growth hormone, progesterone, follicle-stimulating hormone, testosterone (Cusabio, Wuhan, China).

***Quantitative real-time polymerase chain reaction (RT-qPCR) and immunoblot analysis***

For RT-qPCR analysis, total mRNA of the ovaries was extracted by TRIzol (Invitrogen, USA). cDNA was obtained by using a FastQuant RT Kit (Tiangen, China) and was subsequently used for RT-qPCR analysis with SuperReal PreMix Plus (SYBR Green) (Tiangen, China) following the two-step reaction program.

For immunoblot analysis, total protein was extracted from ovaries by RIPA lysis buffer (Beyotime Biotechnology), and the concentration was calculated by the bicinchoninic acid (BCA) method. Samples were separated on 10% SDS-polyacrylamide gels and transferred to polyvinylidene fluoride (PVDF) membranes. Antibodies against the following proteins were used: SIRT6, GAPDH, NF-κB, Ac-NF-κB, (Cell Signaling Technology, USA); TNF-α (Abcam, UK); LLH1 (Santa Cruz Biotechnology, USA).

***Primers used in this research***

Table1. Primers used for RT-qPCR analysis.

| **Gene name** | **Primer sequences** | |
| --- | --- | --- |
| *Sirt6* | forward | 5’-ATGTCGGTGAATTATGCAGCA-3’ |
|  | reverse | 5’-GCTGGAGGACTGCCACATTA-3’ |
| *Plod1* | forward | 5’-GGAGGCTTCATTGGTTATGCC-3’ |
|  | reverse | 5’-GCTGATATTGATTTGCTCCCTCT-3’ |
| *Cyp11a1* | forward | 5’-AGGTCCTTCAATGAGATCCCTT-3’ |
|  | reverse | 5’-TCCCTGTAAATGGGGCCATAC-3’ |
| *Cyp19a1* | forward | 5’-ATGTTCTTGGAAATGCTGAACCC-3’ |
|  | reverse | 5’-AGGACCTGGTATTGAAGACGAG-3’ |

Table2. Primers used for ChIP assay.

| **Gene name** | **Primer sequences** | |
| --- | --- | --- |
| *Cyp11a1* | forward | gtgtgtaggggtgtggtgtg |
|  | reverse | ttCCCCCAACTATCATGCTC |
| *Plod1* | forward | GGTGGAAAGGAGGATGCCC |
|  | reverse | CGTAGAGAGCAAGCAACCTC |
| *Mgarp* | forward | gggtgtcctgaaaatcgctc |
|  | reverse | GGTggtggtagtggcctatg |

**^Fig. Sup.1^**


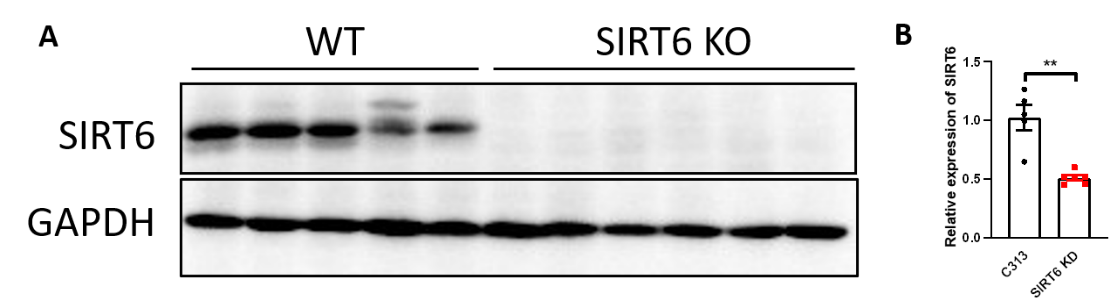


Fig. Sup.1 (A) Western bolt of SIRT6 in ovaries from WT and SIRT6 KO mice. (B) RT-qPCR results of *Sirt6* in GCs cultured for 24 hours.

**^Fig. Sup.2^**


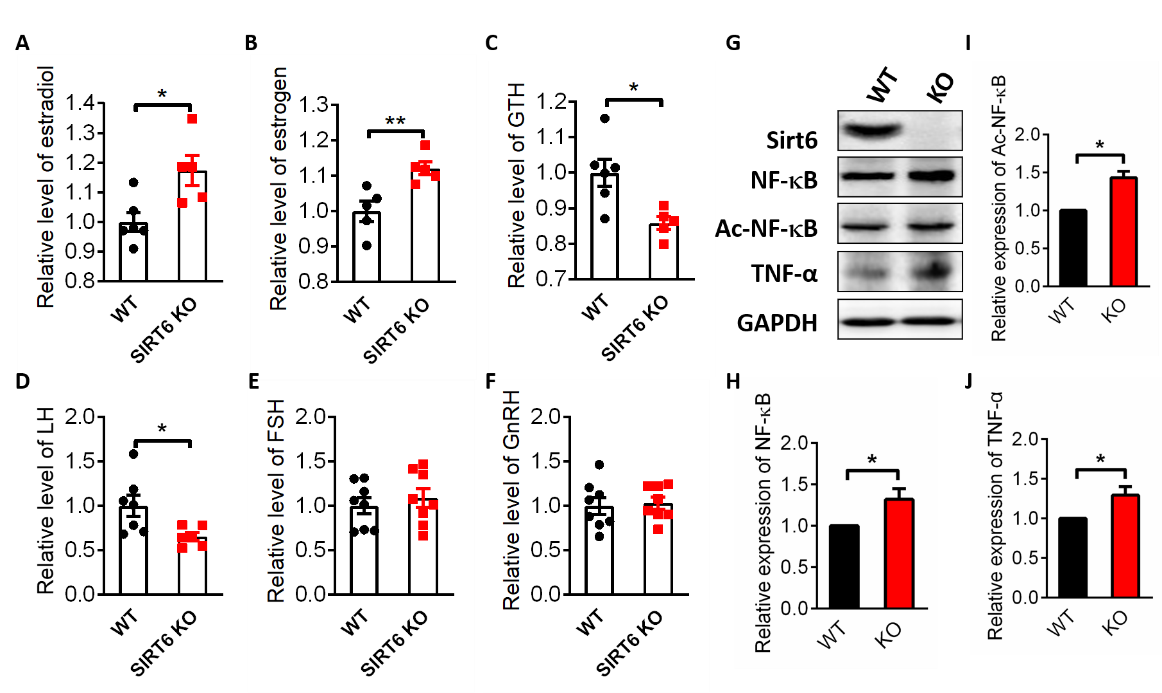


Fig. Sup.2 Comparisons of plasma hormone levels in WT and SIRT6 KO mice. (A) Relative plasma estrogen hormone level; (B) Relative estradiol; (C-F) Relative GTH, LH, FSH, GnRH level in female WT and SIRT6 KO mice. **p*<0.05; ***p*<0.01; ****p*<0.001 (n=5-8 for each genotype). (G-J) Western blot analysis of ac-p65, p65 and TNF-α proteins. **p*<0.05, ***p<0.01*. (n=5-8 for each group).

**^Fig. Sup.3^**


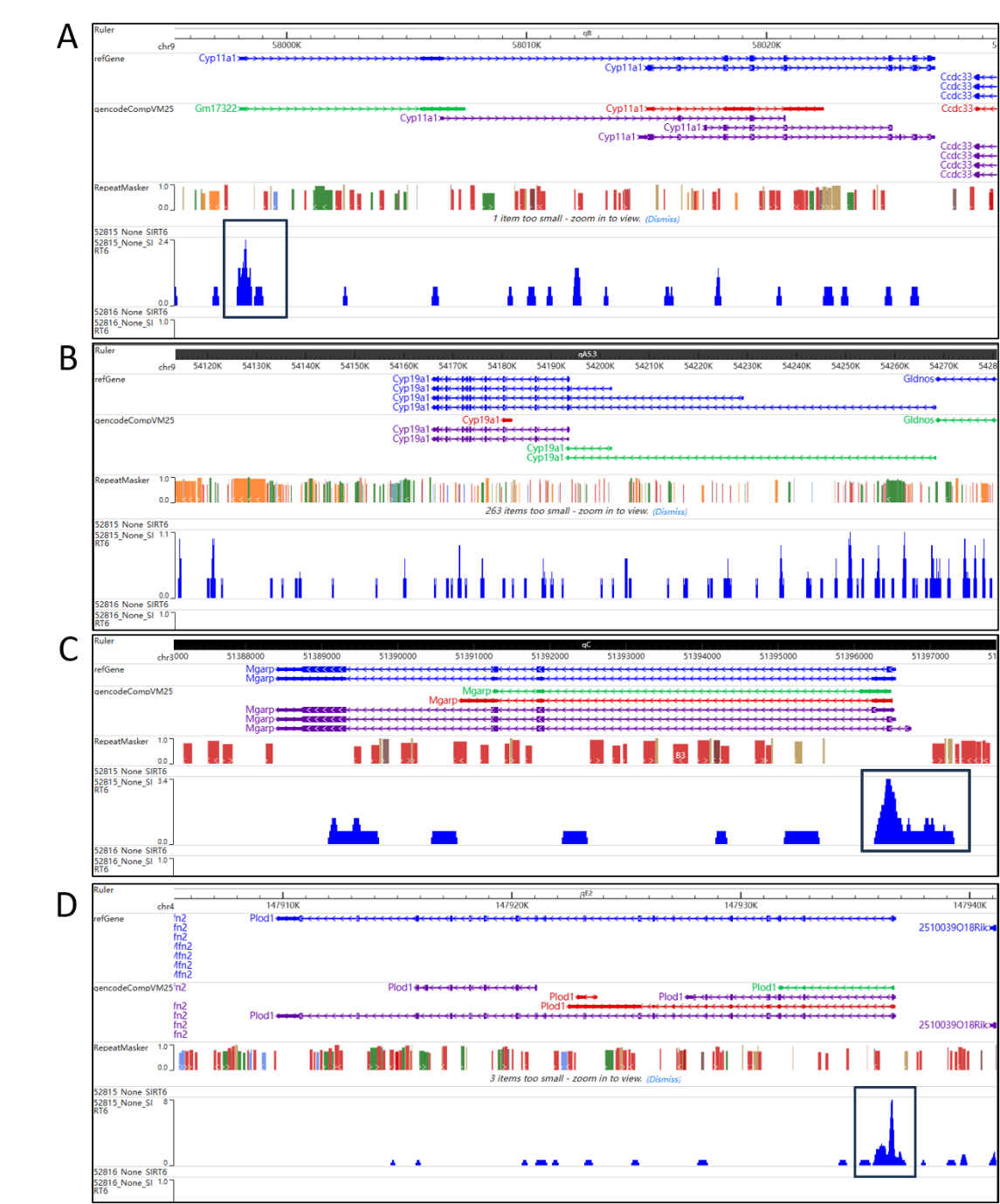


Fig.Sup.3 WashU browser visualization of ChIP-Seq binding profiles of SIRT6 on (A) Cyp11a1, (B) Cyp19a1, (C) Mgarp and (D) Plod1 in mouse embryonic stem cells (data from Cistrome Data Browser). The distribution of ChIP enrichment peaks in the target gene region directly shown at the bottom of the page (marked with black boxes).
